# QuickStats

**Published:** 2015-04-10

**Authors:** FROM THE NATIONAL CENTER FOR HEALTH STATISTICS

**Figure:**
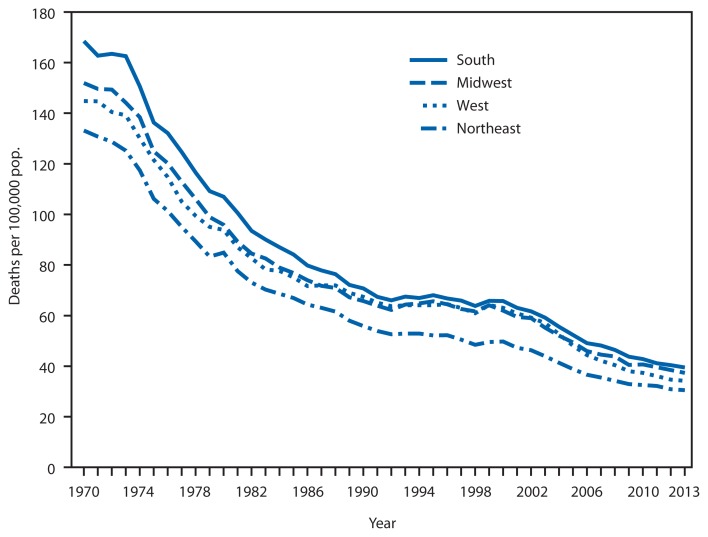
Age–Adjusted Death Rates* for Stroke,^†^ by U.S. Census Region^§^ — United States, 1970–2013 * Per 100,000 standard population. ^†^ Stroke cases are identified using underlying cause of death with codes 430–438 (1970–1998), and I60–I69 (1999–2013) in the *International Classification of Diseases, Eighth, Ninth and Tenth Revisions*. ICD-10 replaced ICD-9 in 1999, and its new classification scheme has had a net effect of increasing counts of stroke as an underlying cause of death by about 6% starting that year. ^§^ *Northeast:* Connecticut, Maine, Massachusetts, New Hampshire, Rhode Island, New Jersey, New York, Pennsylvania, and Vermont; *Midwest:* Illinois, Indiana, Iowa, Kansas, Michigan, Minnesota, Missouri, Nebraska, North Dakota, Ohio, South Dakota, and Wisconsin; *South:* Alabama, Arkansas, Delaware, Florida, Georgia, Kentucky, Louisiana, Mississippi, Maryland, North Carolina, Oklahoma, South Carolina, Virginia, Tennessee, Texas, West Virginia, and District of Columbia; *West:* Alaska, Arizona, California, Colorado, Hawaii, Idaho, Montana, Nevada, New Mexico, Oregon, Utah, Washington, and Wyoming.

The age-adjusted death rates for stroke in all U.S. Census regions in the United States generally decreased from 1970 to 2013, although the rates in all regions were relatively stable from 1992 to 1999. From 1970 to 2013, the rate decreased an average of 3.3% per year in the South, 3.2% in the Midwest, 3.3% in the West, and 3.4% in the Northeast. Throughout the period, the rate was the highest in the South and lowest in the Northeast region.

**Source:** National Vital Statistics System. Mortality public use data files, 1970–2013. Available at http://www.cdc.gov/nchs/data_access/vitalstatsonline.htm.

**Reported by:** Jiaquan Xu, MD, jax4@cdc.gov, 301-458-4086.

